# Domain-specific osmoadaptation revealed by metatranscriptomic analysis in hypersaline environments

**DOI:** 10.1038/s41598-025-04148-4

**Published:** 2025-07-02

**Authors:** Salvador Mirete, María Lamprecht-Grandío, Carolina González de Figueras, José Eduardo González-Pastor

**Affiliations:** 1 Centro de Astrobiología (CAB), CSIC-INTA, Ctra. de Ajalvir km4, 28850 Torrejón de Ardoz, Madrid, Spain; 2 Universidad Atlántico Medio, Ctra. de Quilmes, 37 Tafira Baja, 35017 Las Palmas, Spain

**Keywords:** Metagenomics, Microbiology, Microbial ecology, Astrobiology

## Abstract

High-throughput RNA-seq enables the analysis of gene expression in complex, culture-independent microbial communities. In this study, we used this approach to explore the microbial adaptation to salinity changes in hypersaline environments using samples collected from the Santa Pola ponds (Alicante, Spain). Two metatranscriptomic experiments were conducted: (i) salt concentration from 20 to 30%, mimicking summer evaporation, and (ii) dilution from 30 to 25%, simulating rainfall. As a result, these two experiments revealed significant differences in the gene expression of several metabolic pathways and essential processes, revealing different adaptation strategies between Archaea and Bacteria. Under saline concentration, most bacterial taxa, excluding *Salinibacter*, exhibited higher transcriptional repression compared to Archaea, suggesting an energy conservation response to osmotic stress. In contrast, Archaea maintained metabolic activity, with *Haloquadratum* showing more gene induction than repression, allowing for osmoadaptation. Conversely, in the dilution experiment, Archaea displayed greater transcriptional plasticity than Bacteria, reflecting their ability to dynamically adapt to fluctuating salinity. Further, metatranscriptomics coupled with the analysis of isoelectric point (pI) distributions revealed a remarkable repression of high-pI predicted proteins under high-salt concentration, particularly in Bacteria, suggesting a selective downregulation of basic proteins in response to osmotic stress and highlighting a potential adaptive mechanism. Overall, these findings underscore domain-specific osmoadaptation strategies to cope with osmotic stress in hypersaline environments.

**Supplementary Information:**

The online version contains supplementary material available at 10.1038/s41598-025-04148-4.

## Introduction

High salt content is among the harshest conditions on Earth by which living cells are subjected to extreme osmotic pressure conditions. Hypersaline habitats include saline lakes, solar salterns and sediments where evaporation processes can eventually lead to salt saturation in the form of halite. One of the most emblematic and ecologically informative hypersaline ecosystems is the Santa Pola solar salterns (Alicante, eastern Spain), where natural evaporation and anthropogenic management generate a stable salinity gradient. These salterns have been widely recognized as a model system for studying halophilic microbial communities due to their accessibility, well-characterized physicochemical parameters, and documented microbial diversity^1^. Previous studies in this environment have provided extensive insights into the taxonomic composition of its microbial assemblages, highlighting the dominance of Euryarchaeota, particularly *Haloquadratum walsbyi*, along with *Salinibacter ruber*^2^. Despite its extreme salinity, there are a number of microbial communities thriving in salt-enriched habitats containing over 30% (w/v) total salts^3,4^. Microorganisms thriving under these salinities can be found across several phylogenetic groups belonging to the three domains of life^5^; and they deal with the presence of high salt content and maintain the osmotic balance with the surrounding medium by using two main different strategies: the “salt-in” strategy and the “compatible solutes” or “salt-out” strategy”^6^. The first strategy implies the accumulation of salt inside the cell and, as a result, proteomes in these microorganisms undergo an extensive change in their structures, which involves the over-representation of highly acidic amino acids as well as the presence of a low proportion of hydrophobic residues. Thus, the proteome analysis from halophiles show a lower isoelectric point when compared with non-halophiles^7,8^. Among the microorganisms that use this method to cope with high salt content are several halophilic organisms, such as the bacterium *Salinibacter ruber* and the archaeal *Halobacterium* spp.^9^. The second strategy involves the intracellular accumulation of organic osmotic solutes—such as glycine betaine, glycerol, ectoine, or trehalose—which are compatible with cellular metabolism and do not interfere with essential enzymatic functions. These solutes help the cell maintain osmotic balance with the external hypersaline environment without requiring structural modifications to intracellular proteins^10,11^. The “compatible solutes” strategy is widely distributed across domains, and it does not entail structural modifications of intracellular proteins. These survival mechanisms have profound implications for astrobiology, as they offer a model explaining how microbial life could persist in extraterrestrial environments with high salinity, such as the subsurface oceans of Europa or the chloride-rich brines on Mars^12–14^.

Despite the recent advancements in understanding the mechanisms of salt adaptation, the majority of existing knowledge is derived from studies of cultivated microorganisms or their sequenced genomes. However, this approach detects only a small fraction of the overall functional diversity and thus may overlook specific strategies of adaptation from uncultured microorganisms within a microbial community^15,16^. In hypersaline environments, several new phylogenetic lineages and novel genes without cultured representatives have been detected by using different metagenomics-based methodologies^9,17^. Nevertheless, to date, most metagenomics analyses have been focused on describing the microbial diversity and community structure present in hypersaline environments, whereas only a few have attempted to explore the functional adaptation behind salt perturbations^18–20^. Therefore, an uncultured-approach is instrumental to unveil the different microbial adaptations to high salt content disturbances in the environment. A key strength of this study lies in its metatranscriptomic design, which allows for the direct assessment of gene expression dynamics within active microbial communities in hypersaline environments. Unlike metagenomic approaches that provide a static inventory of genetic potential—including DNA from “dead” or lysed cells—metatranscriptomics captures the real-time functional activity of microorganisms, offering a high-resolution snapshot of in situ physiological responses. This is particularly advantageous in extreme environments, where microbial turnover may be high and relic DNA can confound interpretations of community function. To our knowledge, the application of this approach to hypersaline ecosystems remains rare, making this study the first to explore transcriptionally mediated adaptation strategies under salt concentration and dilution stresses. By linking gene expression profiles to specific taxonomic groups, this method provides deeper insight into the differential roles of archaea and bacteria in osmoadaptation and highlights functionally relevant biosignatures that may otherwise be missed by DNA-based surveys alone.

The solubilized salt amount present in a given ecosystem can be considered as a dynamic parameter that drives the community structure^21,22^. Thus, it is well known that heavy rains as well as strong solar exposure can lead to rapid dilution or concentration of salts in brines, respectively^23–25^. These rapid environmental changes may alter not only the bacterial community composition and abundance but also impact the metabolic activity of that community as a whole. For example, significant changes in microbial and gene abundance were observed at the DNA level when a osmotic shock was applied^18,19,26^. Also, in the Atacama Desert, the driest desert in the world, it was observed that the microbial diversity diminishes as a result of a quick water supply^27^. However, the molecular mechanisms by which microbial communities actively respond to these dynamic environmental changes remain largely unknown. This knowledge gap is partly due to the inherent challenges of studying microbial life in hypersaline habitats, including difficulties in cultivating key taxa, the complex and fluctuating physicochemical properties of brines, and the relative scarcity of in situ functional studies. Moreover, the extreme nature of these environments has historically limited their inclusion in large-scale omics-based monitoring efforts. Yet, understanding how life persists under such extreme osmotic stress is crucial—not only for advancing microbial ecology but also for informing the search for life beyond Earth, where hypersaline niches may represent some of the most likely extraterrestrial habitats^12^. As a result, functional studies in hypersaline systems are rapidly emerging as a critical frontier in both environmental microbiology and astrobiology^14^.

In the present study, a metatranscriptomic survey was conducted with the aim to address the main transcriptional responses that may lead to osmoadaptation of microorganisms when sudden salt changes occur. To this end, a concentration and a dilution experiments were performed in the laboratory by using two different brine samples from the solar salterns of Santa Pola (Alicante, east Spain); and the difference in gene expression was confronted between both situations. The knowledge gained from this study may provide further insights into the molecular adaptive mechanisms employed by microorganisms to cope with perturbations in salt concentration.

## Materials and methods

### Sample and experiments description

Two water samples, denoted as CCAB and C071, were collected on September 7, 2016, from distinct evaporation ponds within the Santa Pola solar saltern system (Alicante, eastern Spain; GPS coordinates 38°11′45.3″N 0°35′52.7″W). This multi-pond saltern complex is composed of a series of shallow basins where seawater undergoes sequential evaporation, creating a gradient of increasing salinity from seawater levels to near-saturation. CCAB was sampled from a high-salinity crystallizer pond (30.4% salinity), while C071 was collected from a lower salinity concentrator pond (20% salinity), reflecting the natural range of salt concentrations found within the saltern system. The salinity was determined using a hand refractometer. 1 L of each sample was collected using sterile plastic jars.

**Fig. 1:**
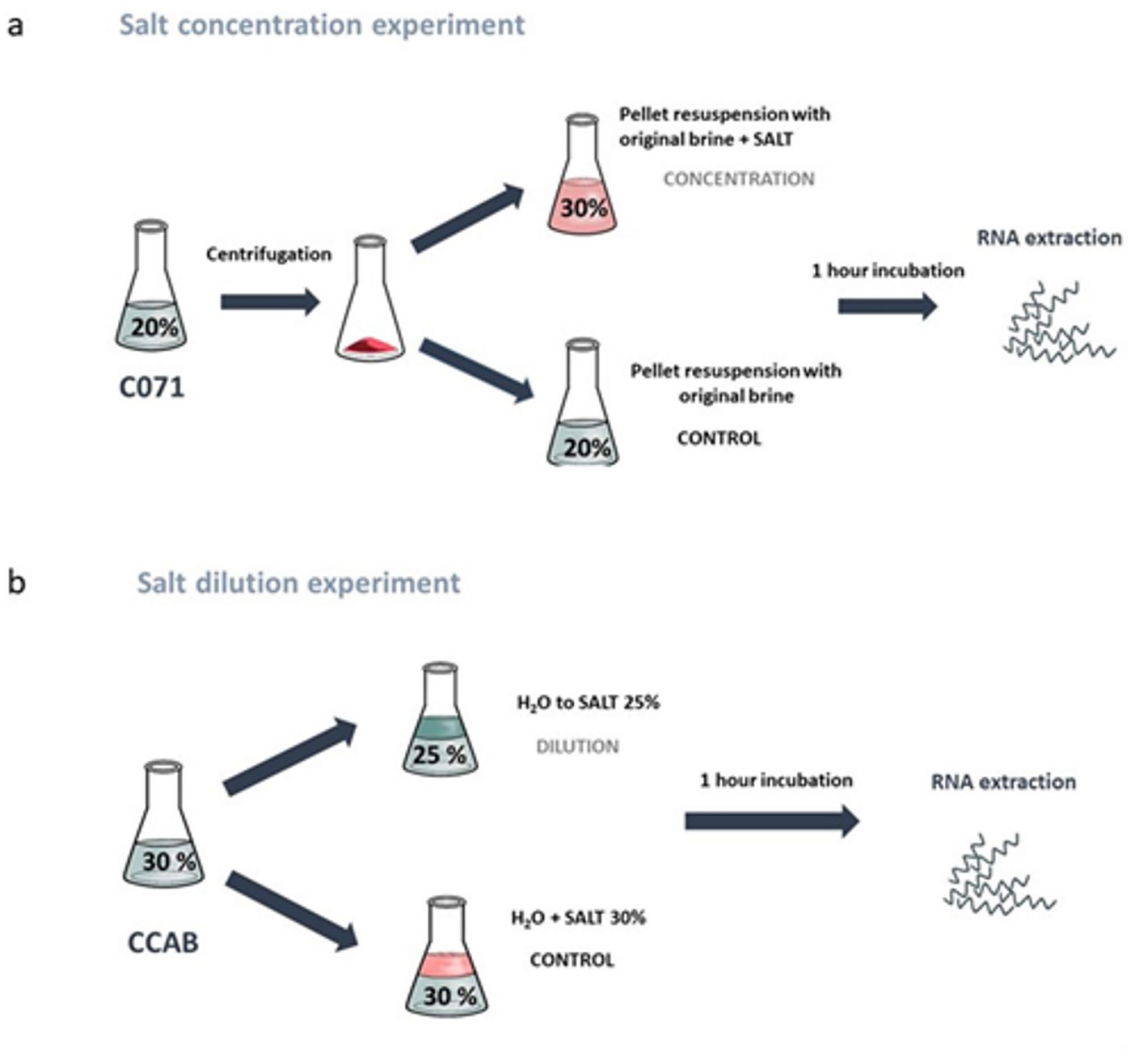
(**a**) Water salt concentration experiment outline. The experiment using the C071 brine (20% salt) was concentrated to 30% salt by centrifuging the cells and resuspending in 30% NaCl solution. (**b**) Water salt dilution experiment outline. The experiment using the CCAB brine (30.4% salt) was salt diluted with the original brine water and adding extra water to dilute the salinity to 25%.

For the concentration experiment, one litre of CO71 solution (20% NaCl) was filtered through sterile cloth using a stainless-steel strainer to remove particulates. The filtrate was distributed into four 250 mL corning tubes and centrifuged at 4000 rpm for 45 min at 20 °C. To further clarify the supernatant, it was centrifuged under the same conditions in three successive rounds in 50 ml Falcon tubes. After the final spin, the clarified supernatant (final volume: 924 mL; OD₆₀₀ = 0.003) was collected, while the pellet was resuspended in 32 mL of the cell suspension. The 924 mL of clarified supernatant was divided into two 450 mL aliquots. One aliquot was supplemented with NaCl (Sigma-Aldrich) to attain a final salt concentration of 30%; the other aliquot served as control (no additional NaCl). Each aliquot was further subdivided into 150 mL portions, which were incubated at 30 °C for 30 min in open beakers to equilibrate temperature and salt concentration. Induction was initiated by adding 5 mL of the 32 mL cell suspension to each 150 mL aliquot. Following a 1 h incubation at 30 °C, the 150 mL mixtures were centrifuged at 10,000 rpm for 20 min at 4 °C using 50 mL Falcon tubes. Pellets from each aliquot were pooled, resuspended, and split into two 1.5 mL microcentrifuge tubes. These were then centrifuged at 10,000 rpm for 15 min at 4 °C. The final pellets were flash-frozen in dry ice and stored at − 80 °C until further analysis. A scheme of the experiment setup is shown in Fig. 1a.

For the dilution experiment, we used CCAB samples with an initial salt concentration of 30.4%. Experimental samples were diluted to 25% NaCl by adding Milli-Q water, while control samples were diluted with an equivalent volume of SW30 (30% NaCl solution) to maintain constant salinity. This approach allowed for a direct comparison between dilution-induced stress and unchanged salinity conditions under identical volumetric manipulations. The final salinity of 25% was deliberately selected to avoid excessively harsh osmotic stress that could compromise microbial viability or suppress transcriptional activity. Extreme halophiles are known to be highly sensitive to sharp reductions in salinity^28^, and we sought to ensure that the microbial community remained metabolically active to allow for robust metatranscriptomic analyses. Following dilution, both experimental and control samples were incubated at room temperature (∼22–24 °C) for 1 h. Subsequently, 350 mL aliquots were collected from each condition and centrifuged at 10,000 rpm for 15 min at 4 °C. The resulting cell pellets were flash-frozen in dry ice and stored at − 80 °C until further analysis. A scheme of the experiment setup is shown in Fig. 1b.

For each sample, three replicates were processed.

### Extraction of nucleic acids

Microbial cells for DNA extraction were collected from brine samples by filtration on a 0.22-μm-pore-size membrane filter (Nalgene). DNA was extracted from these filters by using a CTAB method^29^. Briefly, the filters were incubated in a lysis buffer (Tris–HCl, EDTA, Na₂HPO₄, SDS) at 65 °C, followed by centrifugation to recover cell lysates. Supernatants were treated with NaCl and CTAB, incubated, and subjected to phenol–chloroform-isoamyl alcohol extraction. DNA was precipitated using isopropanol, washed with 70% ethanol, and pelleted by centrifugation. The resulting DNA pellet was air-dried and resuspended in sterile deionized water.

RNA was extracted from the cell pellets with the RNeasy PowerSoil Total RNA Kit (QIAGEN; catalog number 12866–25) and DNA was removed using the RNase-Free DNase Set (Qiagen) following manufacturer’s instructions. Both DNA and RNA samples were sent to Sistemas Genómicos S.L. (Valencia, Spain) for high-throughput sequencing.

The quality and the quantity of the total DNA was determined in Nanodrop-1000 and by agarose gel electrophoresis. The quality and the quantity of the total RNA has been determined in Bioanlayzer 2100 (RNA 6000 Nano chip assay) and Qubit 3.0 (Quant-It dsRNA BR Assay).

### DNA Libraries preparation and sequencing

DNA libraries were constructed with the TruSeq DNA Sample Kit following Illumina´s protocols and recommendations. Briefly, DNA was fragmented in Covaris. The DDN fragments then went through an end repair process, the addition of a single ‘A’ base to the 3’ end and then ligation of the indexed- adapters. The quality of the libraries was analyzed in TapeStation 4200, High Sensitivity assay; the quantity of the libraries was determined by real-time PCR in LightCycler 480 (Roche).

Prior to clusters generation pooling of the libraries was performed. The pool of the libraries was sequenced by paired-end sequencing (100 × 2) in Illumina Hiseq 2500 sequencer (Illumina Inc, San Diego, CA, USA). A total of 76.3 and 60.6 million sequenced reads were generated for C071 and CCAB samples, respectively.

### cDNA Libraries preparation and sequencing

Total RNA was rRNA depleted using Ribo-Zero™ rRNA Removal Kit Bacteria (Illumina) and cDNA libraries were constructed using the TruSeq RNA Library Prep Kit (Illumina) following Illumina´s recommendations at Sistemas Genómicos. We started from 2 μg of total RNA (RIN > 9) libraries. Briefly, RNA ribosomal depleted was chemically fragmented prior to reverse transcription and cDNA generation. The cDNA fragments then went through an end repair process, the addition of a single ‘A’ base to the 3’ end and then ligation of the adapters. Finally, the products were purified and enriched with PCR to create the indexed final double stranded cDNA library.

The quality of the libraries was analyzed in TapeStation 4200, High Sensitivity assay; the quantity of the libraries was determined by real-time PCR in LightCycler 480 (Roche).

The pool of libraries was sequenced (100 × 2) in Illumina HiSeq 2500 sequencer. Sequencing readings were paired-end with a length of 101 bp reading performed in 6 samples. The estimated coverage was around 30 million reads per sample (1 lane). Library generation and RNA sequencing was done at Sistemas Genómicos S.L. (Valencia, Spain) following manufacturer´s instructions.

The metagenomics assembly results and the total number of sequenced reads for each metatranscriptomic sample and replica for each sample are summarized in Table S3.

### Metagenomic sequencing, bioinformatic analysis and gene annotation

The quality of the raw data was checked using FASTQC tools. The adaptors were removed using the Fastq mcf (v1.04.803)^17^ tool. A quality filter was made with Cutadapt (v1.9.1)^30^ using a quality window value of 30 and then the paired-end reads were merged using Flash (v1.2.11)^31^. Another step was needed to mask the low quality bases. The assembler used was Megahit^32^. A list of several k-mers was used, with sizes from 15 to 99. The gene detection was made using Glimmer3 ^33,34^.

For taxonomic assignation, these ORFs were annotated with Blast 0.2.2.30 + ^35^ with an E-value cutoff of 1e- 3 against the latest version (UniProtKB/Swiss-Prot Release 2015_08) of the Uniprot Swissprot protein curated database for Bacteria (http://www.uniprot.org/). All the small local alignments were removed applying a filter requiring an alignment size of at least the half size of the smallest sequence.

The sequences without a hit were annotated using BLAST V.2.2.30 + against the NT database (non-redundant nucleotide sequences from all traditional divisions of GenBank, EMBL, and DDBJ excluding GSS, STS, PAT, EST, HTG, and WGS) from the NCBI. All the small local alignments were removed.

Identified genes were functionally annotated using the functional annotation of Uniprot^36^ database for the three main functional categories (biological process, molecular function, and cellular component) with associated KEGG Ontology pathways^37^ and gene Ontology database^38^. Lastly PFam^39^ terms were obtained.

Additionally, 16S rRNA gene sequences were extracted from the metagenome datasets by using Barrnap v 0.9. Taxonomic classification was performed within the SILVAngs 1.4 pipeline using BLASTn (v2.11.0 +) with standard parameters against the non-redundant SILVA SSU Ref dataset release^40^. Representative OTU sequences were aligned to the reference database, and taxonomy was assigned based on sequence similarity. Reads with a classification score below the value of 93—calculated as % sequence identity + % alignment coverage)/2—were considered unclassified and grouped as “No Relative”.

Normalization of the data presented in the bar plots was performed in Microsoft Excel using relative abundance values from Supplementary Tables S1 and S2. All bar charts were generated in Excel based on these normalized datasets.

Isoelectric points for each dataset were predicted with the icp-protein algorithm implemented in ICP 2.0^41^ using amino acid predicted sequences with provided fasta files.

### RNA transcriptomics analysis

The quality control of the raw data was performed using the FastQC v0.11.4 tool. Then, the raw paired-end reads were mapped against the previously de-novo assembled genome using Bowtie2 v2.3.1^42^. Insufficient quality reads (phred score < 1) were eliminated using Samtools 1.2^43^ and Picard Tools 2.12.1.

Expression levels were calculated using the HTSeq^44^.This method employs unique reads for the estimation of gene expression and filters the multimapped reads. Differential expression analysis between conditions was assessed using DESeq2^45^. Finally, we selected differentially expressed genes with a P value adjusted by FDR^46^ < 0.05 and a fold change (FC) of at least 2. The DEG analysis between different groups was done using statistical packages designed by Python and R. Using DESeq2 algorithm^45^ applying a differential negative binomial distribution for the statistics significance^46^ we identified genes differentially expressed. We considered as differently expressed genes those with a FC value below − 2 or higher than 2 and with P value (Padj) corrected by FDR ≤ 0.05 to avoid identification of false positives across the differential expression data. A total of 6,908 out of 8,740 sequences from C071 (Table S1) and 2,985 out of 5,911 from CCAB (Table S2) exhibited significant differential expression (FC > 2 and -2 < ; *p* < 0.05).

## Results and discussion

### Metagenomic and metatranscriptomic sequencing statistics

Two experiments were accomplished from two brine samples, named CCAB and C071 from Santa Pola (Alicante, Spain), with different salt concentrations of 30.4% and 20%, respectively. Conducting comparative experiments on natural settings is challenging because microbial diversity is frequently uneven. This is especially true in hypersaline environments where salt concentration and UV radiation exert strong selective pressures on microbial communities^47^. However, our design based on using two samples with different salt concentrations enables the assessment of transcriptional and functional adaptations under distinct osmotic stresses while controlling for broad taxonomic background. Therefore, despite subtle community differences, the similar overall taxonomic structure provides a framework to decipher domain-specific adaptation mechanisms to osmotic stress. This approach is consistent with previous metagenomics studies showing that functional resilience often outweighs exact species composition in response to salinity disturbances^48,49^.

Owing to the lack of a reference genome associated to the hypersaline samples studied, the metatranscriptomic reads of each sample were mapped against a metagenome assembled for each corresponding sample obtained in order to identify genes with differential expression. Only those reads that hybridized in silico between the metatranscriptome and the metagenome were taken into account.

The percentage of mapped High Quality (HQ) reads ranged from 33 to 60%. Genes derived from the assembled metagenomes were annotated in the contigs obtained and the microbial community composition analysis was based on the taxonomic assignment of the 16S rRNA and nucleotide gene sequences at the phylum- and genus-level. This latter taxonomic level was selected for comparison because of its ecological relevance^50^.

### Microbial adaptations to salt shocks

**Fig. 2:**
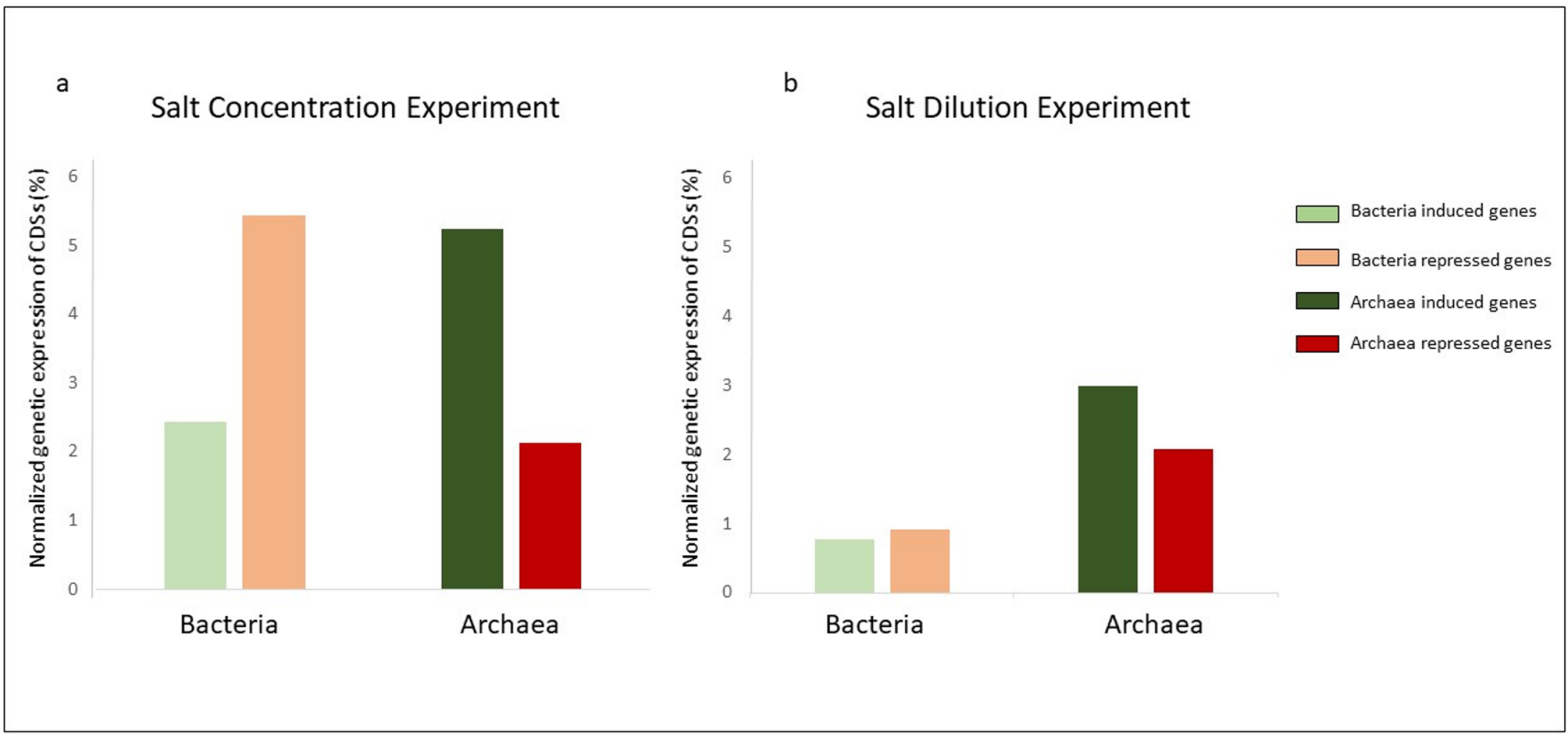
Bacterial and archaeal population significantly expressed after (**a**) salt concentration and (**b**) dilution experiments normalized by the total from sequenced genes. The standardization was performed as the percentage of number of genes differential expressed partitioned by the number of genes assigned to Archaea or Bacteria.

Microbial communities responded to salt fluctuations through distinct transcriptional adjustments. A 10% salt increase led to a 5.4% repression in bacterial gene expression whereas archaeal transcription increased by 5.2% (Fig. 2a). In contrast, the dilution experiment triggered a more pronounced transcriptional response in Archaea than in Bacteria. Archaea induced 2.9% and repressed 2.07% of their genes, while bacterial induction and repression were minimal (0.7% and 0.9%, respectively) (Fig. 2b).

### Microbial adaptations to salt concentration

**Fig. 3:**
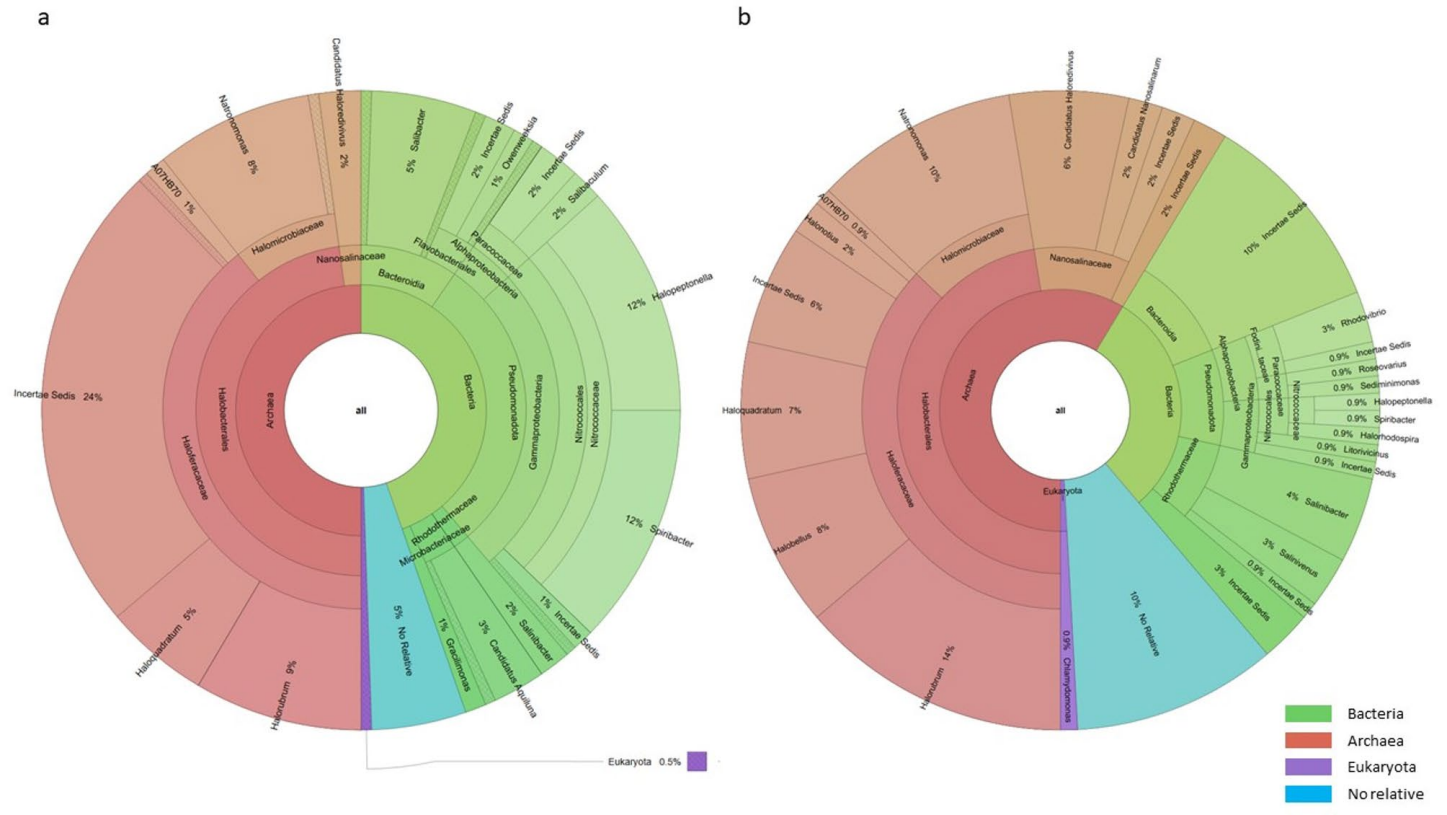
Krona charts of the 16S rRNA gene sequences showing different taxonomic levels detected from (**a**) C071 and (**b**) CCAB samples.

**Fig. 4:**
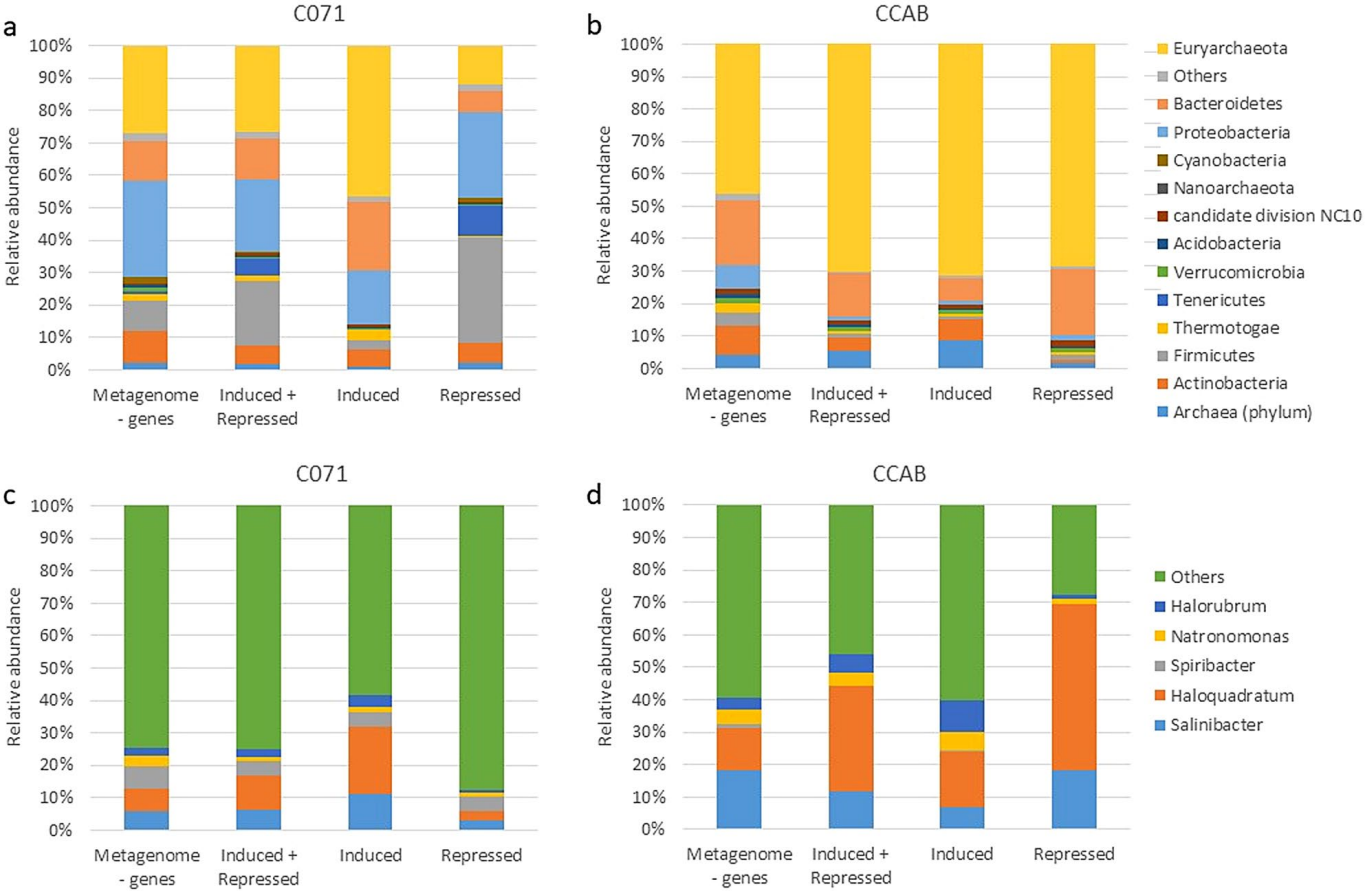
Microbial diversity breakdown based on the gene annotations at the phylum level from (**a**) the C071 sample (concentration experiment) and (**b**) the CCAB sample (dilution experiment); and at the genus level from (**c**) the C071 sample (concentration experiment) and (**d**) the CCAB sample (dilution experiment). Only those phyla representing more than 1% in at least one dataset were drawn at the phylum level and 10% at the genus level.

To our knowledge this study represents the first metatranscriptomic survey examining the functional activity and adaptive responses of saltern-associated microbial communities to salt shocks. In the salt concentration experiment, a 10% salt increase induced substantial taxonomic shifts and widespread transcriptional reprogramming, revealing distinct strategies between Archaea and Bacteria in response to extreme osmotic stress (Figs. 3a and 4a). Therefore, Euryarchaeota remained the dominant phylum in the concentrated C071 sample, representing approximately 50% of the 16S rRNA sequences and 27.13% of the assigned genes (Figs. 3a and 4a). Euryarchaeota exhibited high transcriptional plasticity, with 26.49% of its genes differentially expressed. Notably, gene induction (46.57%) far exceeded repression (11.80%), highlighting the capacity of halophilic Archaea, particularly *Haloquadratum*, for osmoprotection and energy generation^5,51^. In contrast, Proteobacteria and Bacteroidetes showed more mixed responses. Proteobacteria (29.26% of 16S rRNA, 29.77% of genes) exhibited a more repressive transcriptional profile overall (with 26.49% of genes repressed versus 16.38% induced), suggesting that many bacterial taxa belonging to this group adopt an energy-conserving strategy under salt stress. For example, key bacterial replication-related genes and ribosomal proteins were downregulated—141 bacterial ribosomal transcripts were repressed (0.22%)—indicating a slowdown in growth and protein synthesis. Meanwhile, Bacteroidetes (2% of 16S rRNA, 12.03% of genes), was mainly represented by the genus *Salinibacte*r whose taxonomy has been updated recently and is now classified under the phylum Rhodothermaeota^52^. In our data, however, *Salinibacter* appears to be classified under both Bacteroidetes and Rhodothermaeota (see Fig. 3), which explains its presence under multiple classifications. In *Salinibacter,* induction (11.14%) far exceeded repression (2.83%), suggesting an efficient metabolic response to extreme salinity. These results are in line with previous studies identifying *Salinibacter ruber* as a key player in hypersaline environments, where it optimizes metabolic pathways to withstand osmotic stress^53^.

**Fig. 5:**
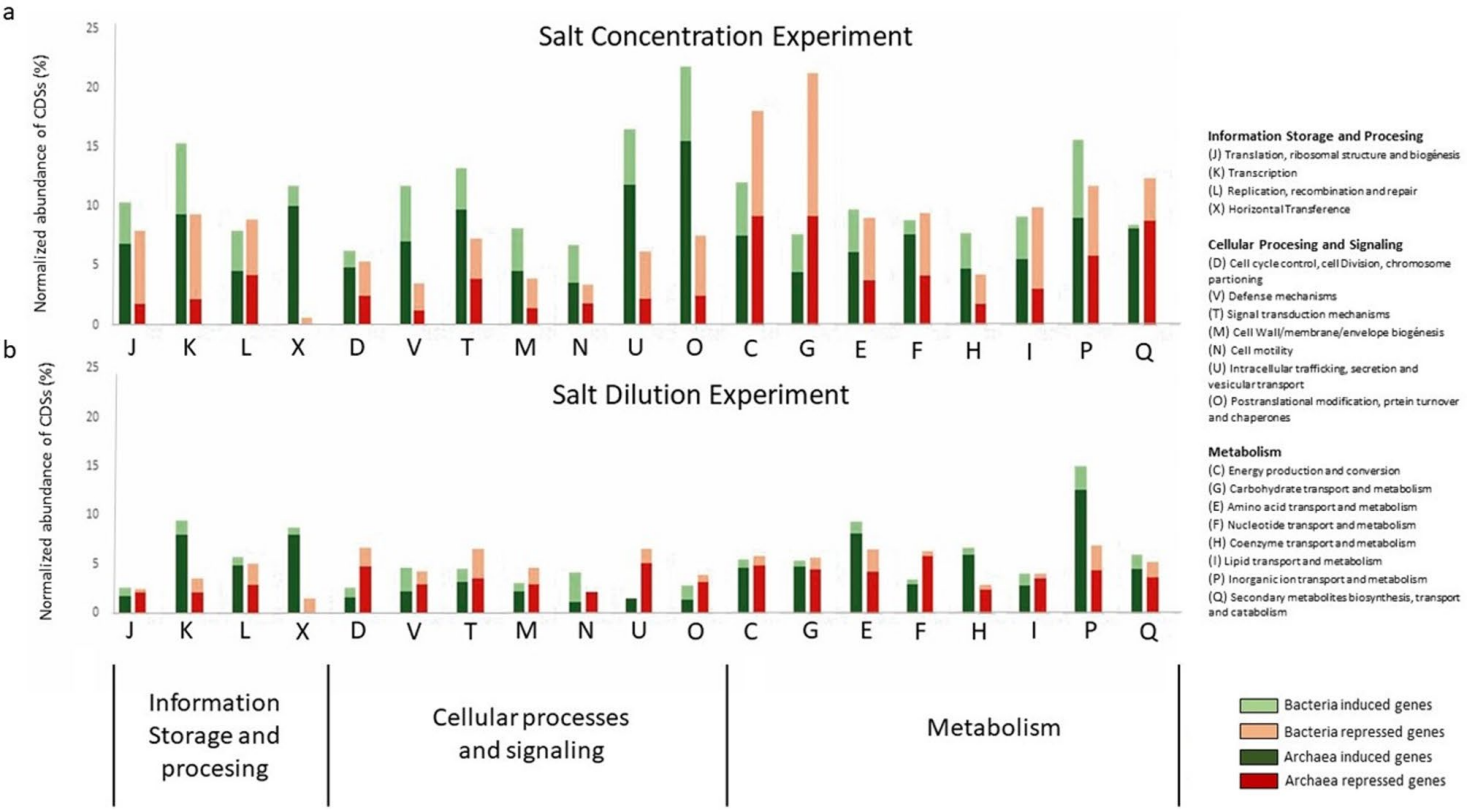
COG (Clusters of Orthologous Groups) categories. Percentage of significantly induced or repressed CDSs from total sequenced genes belonging to Clusters of Orthologous Groups (COG) for (**a**) salt concentration experiment and (**b**) salt dilution experiment. The standardization was performed as the percentage of number of genes differential expressed assigned to each COG partitioned by the total number of genes assigned to each COG.

**Fig. 6:**
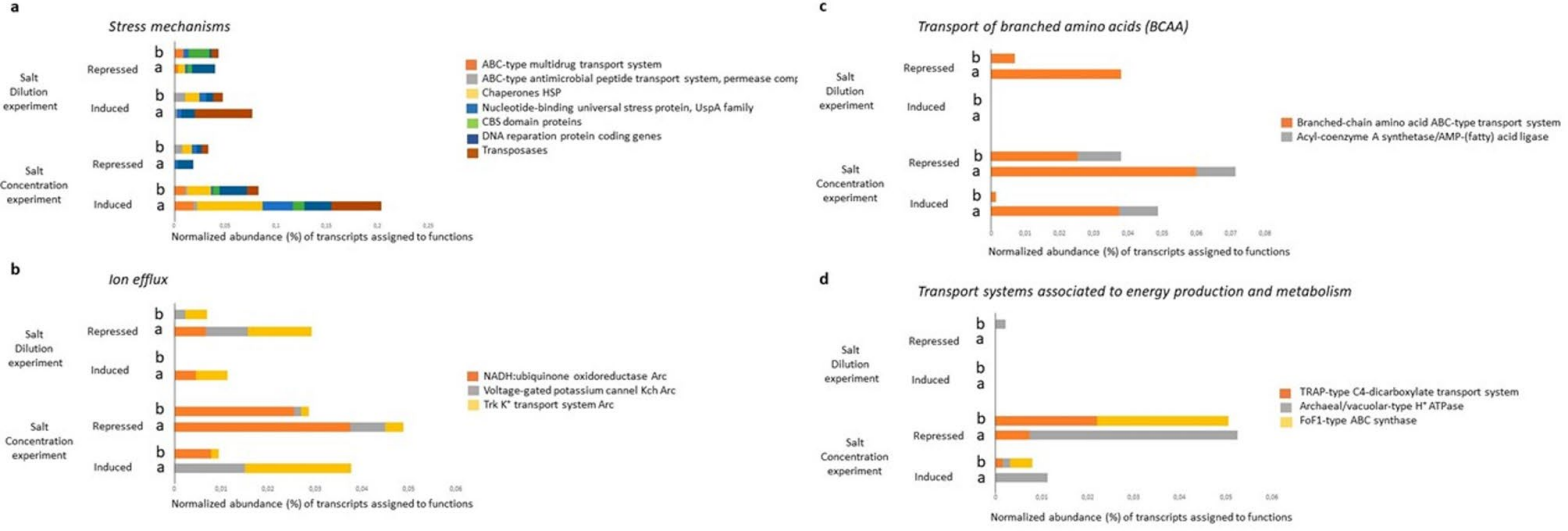
Relative abundance (%) of significantly induced or repressed transcripts associated with stress mechanisms; ion efflux; transport of branched amino acids (BCAA); and transport systems associated to energy production and metabolism of the two experiments (Salt dilution experiment and Salt concentration experiment) from total sequenced genes. The expression is referred to Bacteria (b) and Archaea (a). The standardization was performed as the percentage of number of genes differential expressed partitioned by the number of genes assigned to Archaea or Bacteria.

A central adaptive response to increased salinity involves extensive reprogramming of energy metabolism and stress defense pathways. Comparative analysis of cluster orthologous groups (COGs) (Fig. 5) revealed that post-transcriptional modifications, protein turnover, and chaperones (COG O) were significantly induced in both Archaea (15.47%) and Bacteria (6.23%), likely to aid in protein stabilization under high salt conditions^54^. Heat shock proteins (HSPs), which rank among the top 2% most highly expressed transcripts in Bacteria (Table S4), underscore the reliance on molecular chaperones to maintain proper protein conformation. Besides, universal stress protein A (UspA) genes, known to confer resistance to DNA damage under multiple stressors^55,56^, were induced (Fig. 6a) —particularly in *Haloquadratum*—further reinforcing stress response activation. Moreover, this archaeon maintained active transcription of replication and translation genes, indicative of a strategy aimed at sustaining metabolic and growth activity despite harsh conditions.

Maintaining osmotic balance is critical under extreme salt concentrations, and several COGs highlight the molecular mechanisms involved. For example, intracellular trafficking and vesicular transport (COG U) were significantly upregulated—predominantly in Archaea (11.8%) and to a lesser extent in Bacteria (4.6%) (Fig. 5)—suggesting an enhanced “Salt In” strategy to facilitate the uptake of compatible solutes and ions needed for osmoprotection^57^. Concurrently, inorganic ion transport and metabolism (COG P) showed notable induction in Archaea (8.9%) and Bacteria (6.6%), accompanied by the activation of defense mechanisms (COG V), including ABC-type multidrug transporters, multidrug efflux pump subunit AcrB, and Na⁺-driven efflux systems. Also, potassium homeostasis plays a key role in counterbalancing high extracellular Na⁺ and thus, Archaeal Trk K⁺ transporters (Fig. 6b) were induced in *Haloquadratum*, *Halomicrobium*, and *Haloferax*, facilitating K⁺ uptake as part of the “salt-in” strategy^5,58^. Additionally, the high induction of sulfate permeases in *Salinibacter* from the major facilitator superfamily (MFS) suggests an active expulsion of sulfate and other metabolites to maintain cellular homeostasis^59,60^.

Switching to high salinity also stimulated genetic adaptation mechanisms and as so transposase-related genes (COG X) were significantly induced in Archaea—especially in *Haloferax*, *Halogeometricum*, and *Haloquadratum*—suggesting that horizontal gene transfer and genomic rearrangements contribute to rapid adaptation to extreme osmotic conditions^61–63^. In contrast, bacterial transposase expression was minimal, highlighting distinct evolutionary strategies between both domains.

It is worth to note that not all pathways were activated; many functions seemed deliberately repressed to conserve energy. For instance, the active transport of branched-chain amino acids in Archaea (Fig. 6c) as well as carbohydrate transport and metabolism (COG G) with 9.18% repression in Archaea and 7.4% in Bacteria (Fig. 5) was among the most repressed functions. The latter included downregulation of ABC-type sugar transport systems, ABC-type glycerol-3-phosphate transporters, TRAP-type C4-dicarboxylate transporters and extracellular solute-binding proteins (Fig. 6d and Table S5), reflecting a shift away from energy-intensive metabolic processes. Similarly, energy production and conversion (COG C) were strongly downregulated (9.23% in Archaea, 8.8% in Bacteria), indicating a coordinated reduction in ATP-generating pathways as cells adjust to the osmotic stress environment.

Overall, the salt concentration experiment reveals a complex network of adaptive responses that are deeply intertwined with the microbial taxonomic structure. Archaea, particularly those within Euryarchaeota such as *Haloquadratum* and *Natronomona*s, respond to a 10% salt increase with robust gene induction across multiple functional categories—including ion transport, stress response, and even genetic adaptation—enabling them to sustain metabolic activity under extreme osmotic stress^5,51^. In contrast, bacterial populations adopt a more conservative strategy characterized by widespread repression of growth- and energy-intensive functions, such as replication, translation, and central metabolism, likely to conserve energy under adverse conditions. These findings support the notion that adaptation to environments of persistent energy stress (e.g. high temperature, high salinity and low pH) is the key element that sets Archaea apart from Bacteria^64^. Moreover, this differential strategy is further supported by the induction of specific stress-related genes, such as HSPs in Bacteria, even as they overall downregulate metabolic functions.

The coordinated induction of intracellular trafficking (COG U), inorganic ion transport (COG P), and defense mechanisms (COG V) underscores the importance of maintaining osmotic balance and membrane integrity during salt stress. Moreover, the significant upregulation of transposase-related genes in Archaea (COG X) suggests that genetic plasticity may be a crucial component of their adaptation, enabling rapid evolutionary responses to environmental changes^65^.

### Microbial adaptations to salt dilution

The salt dilution experiment elicited a complex and stratified transcriptional response within the microbial community, reflecting both taxonomic shifts and a dynamic reprogramming of gene expression in response to dilution perturbation. Metagenomic and transcriptomic analyses revealed that under salt dilution, Euryarchaeota dominated the community (56% of 16S rRNA sequences; 46.10% of genes) and exhibited broad transcriptional range, with 70.11% of their genes differentially expressed (Figs. 3 and 4). In contrast, bacterial transcriptional changes were more modest (0.7% induction, 0.9% repression) (Fig. 2), emphasizing distinct adaptive strategies between the two domains. To cope with this salt perturbance in Bacteria, we hypothesized that this gene expression pattern may be balanced with the enrichment of rare osmoregulatory genes based on previous findings during dilution events in *Salinibacter ruber* pangenomes^19^.

**Fig. 7:**
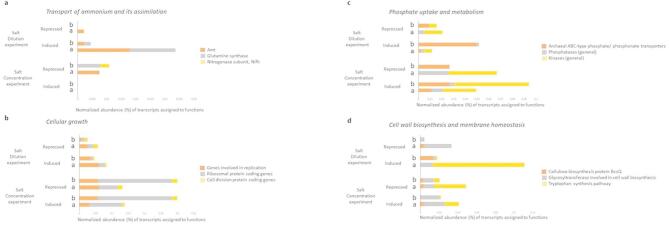
Relative abundance (%) of significantly induced or repressed transcripts associated with transport of ammonium and its assimilation; cellular growth; phosphate uptake and metabolism; and cell wall biosynthesis and membrane homeostasis of the two experiments (Salt dilution experiment and Salt concentration experiment) from total sequenced genes. The expression is referred to Bacteria (b) and Archaea (a). The standardization was performed as the percentage of number of genes differential expressed partitioned by the number of genes assigned to Archaea or Bacteria.

A hallmark of the dilution response is the pronounced induction of nitrogen assimilation pathways (Fig. 7a). Analysis of the top 2% of the most highly expressed transcripts revealed that 10 transcripts were directly linked to nitrogen metabolism and amino acid transport—such as the ammonia channel protein AmtB and Nitrogen regulatory protein PII (Table S6). This induction was predominantly observed in archaeal genera (e.g., *Halobacterium,* *Halorubrum*, and *Haloquadratum)*, with a minor contribution from the bacterial fraction (Fig. 7a). The remarkable activation of these pathways suggests that increased nitrogen uptake is essential for reactivating metabolic functions when external salt concentrations drop, thereby promoting biosynthesis and energy generation^66,67^. Also, this result aligns with recent work on flood-induced metabolomic responses in hypersaline communities, which showed significant enrichment of nitrogen reduction pathways^20^.

The modulation of growth-related functions also characterizes the salt dilution response. A low bacterial fraction of replication-related transcripts (0.03%) were induced, while 0.007% were repressed (Fig. 7b)—an observation largely driven by the halotolerant gram-positive bacterium *Corynebacterium*, which presents an optimal growth rate between 5 and 15% of NaCl^68^ and whose induced replication genes accounted for 73%. In Archaea, replication-related transcripts were induced (0.06%) and repressed (0.02%), with a striking 90% of the repressed genes attributed to *Haloquadratum*. Similarly, bacterial ribosomal protein gene expression was nearly balanced (0.007% induced versus 0.009% repressed). *Corynebacterium* induced ribosomal expression, aligning with the replication rate increase, so this points out that the growth of this genus may be positively affected by salt dilution. In Archaea, overall expression was balanced (0.018% induced and 0.018% repressed) but with *Haloquadratum* showing pronounced repression (81.8% of total repressed transcripts). These contrasting patterns suggest that while some archaeal taxa such as *Halorubrum*, *Halovivax*, and *Natrialba* maintain growth and protein synthesis under dilution, others downregulate replication and translation—likely as an energy-conservation measure in response to reduced salt stress.

Maintenance of osmotic balance under salt dilution is achieved through intricate ion transport and membrane remodeling. In this experiment, ABC-type phosphate/phosphonate transporters were predominantly induced (0.049%) compared to a modest repression (0.009%) (Fig. 7c). These transporters—including PstS, PstB, PhnC, PhnD, and PhnE—were highly expressed in Archaea (e.g., *Halorubrum,* *Halovivax*, *Salinarchaeum*, *Natrialba*, and *Natronomonas*) while being repressed in *Halorhabdus,* reflecting a degree of niche differentiation. Ion homeostasis is further regulated through potassium transport^69^ and voltage-gated K⁺ channels (Kch) were observed to be repressed in both Archaea (0.009%) and Bacteria (0.002%) (Fig. 6b), suggesting reduced K⁺ uptake under dilution. In contrast, the Trk K⁺ transport system exhibited a differential response: archaeal Trk transporters were mostly repressed in genera such as *Halovivax*, *Natronomonas*, and *Haloquadratum* (0.013%), whereas others, including *Halobacterium*, *Haloferax*, and *Salinarchaeum*, showed a modest induction of these transporters (0.007%). Meanwhile, bacterial Trk transporters were exclusively repressed. These observations highlight again the nuanced niche differentiation of intracellular ion balance as the overall community adjust to a lower osmotic environment.

COG analysis further shed light on these adaptive mechanisms. For instance, signal transduction functions (COG T) were among the most significantly repressed under dilution, particularly those associated with CBS and GAF domain proteins in both *Haloquadratum* and *Salinibacter* (Table S7). This suggests that a decrease in external salt may diminish the necessity for certain regulatory responses, thereby streamlining energy expenditure. Additionally, COG E (amino acid transport and metabolism) was prominently overexpressed—specifically, genes involved in tryptophan synthesis (e.g. indole-3-glycerol phosphate synthase) were induced in Archaea (Fig. 7d and Table S6), which is critical for anchoring membrane proteins and restoring membrane stability^70,71^.

The transcriptional response to salt dilution also involves significant modulation of stress-related genes. Notably, ABC-type antimicrobial peptide transporters were induced (0.01%) primarily in *Salinibacter,* which may serve to defend against residual environmental stressors despite the overall decrease in salinity. Additionally, HSPs, which are essential for protein stability under stress^54^ exhibited a polarized response in the dilution experiment; HSP genes were induced exclusively in Bacteria while being repressed in Archaea. This differential regulation implies that, under dilution, bacterial populations rely on HSPs to mitigate protein misfolding, whereas some archaeal groups may shift their strategy away from HSP production.

Collectively, these findings indicate that Archaea and Bacteria employ fundamentally different strategies to cope with salt dilution. Dominant Archaea, particularly within Euryarchaeota, exhibit extensive transcriptional plasticity—upregulating nitrogen assimilation, phosphate uptake, and selective ion transport systems—to rapidly re-establish metabolic equilibrium under decreased salt conditions. Meanwhile, bacterial responses are more subdued; they display limited induction of growth-related and stress-response genes, instead favoring energy conservation strategies. This differential response not only reflect their evolutionary adaptations to hypersaline environments^9,64,72^, but also supports the notion that Archaea are more transcriptionally dynamic under dilution stress, a finding consistent with previous observations^18^.

### Isoelectric point modulation under salt fluctuations

**Fig. 8:**
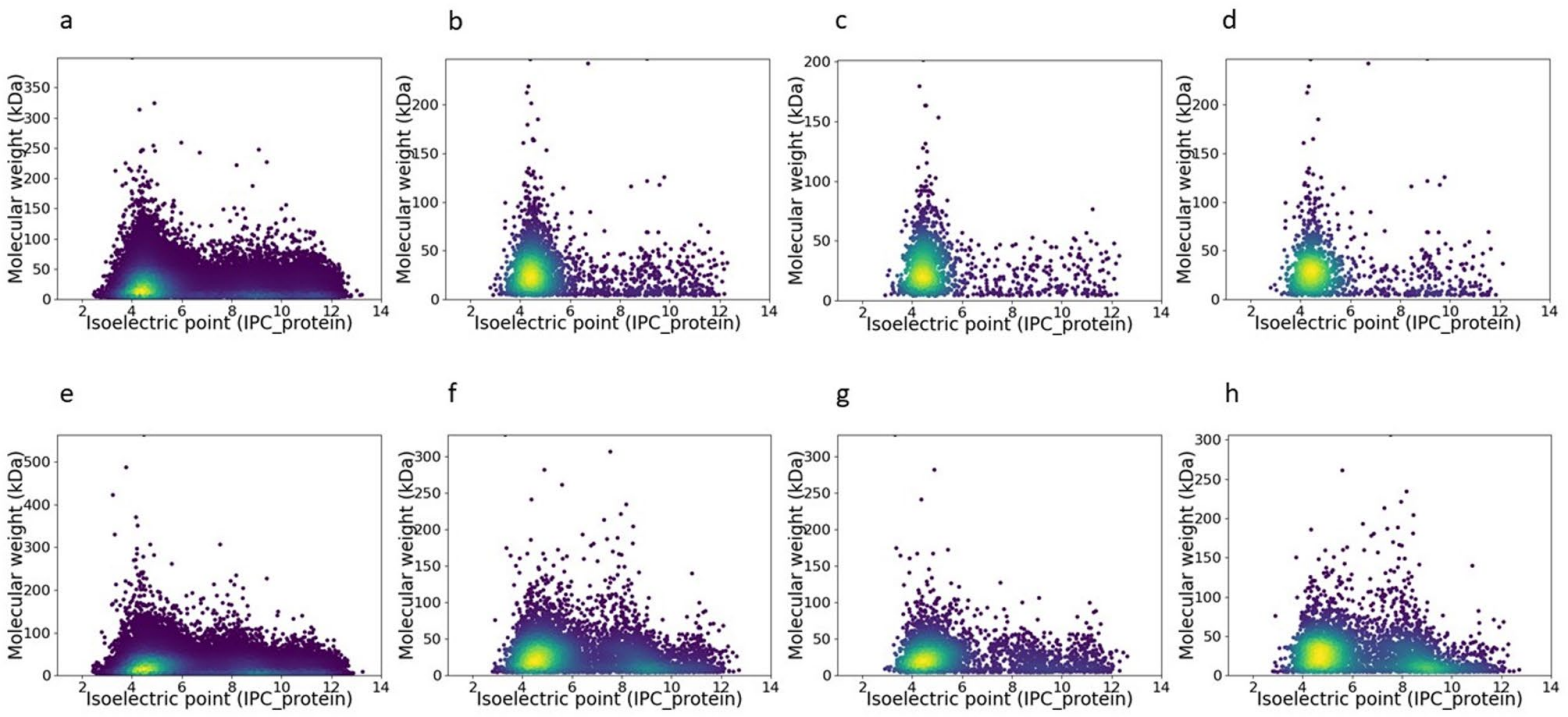
Distribution of predicted proteins based on isoelectric point (pI) and molecular weight under salt concentration (C071) and dilution conditions (CCAB). Panels (**a**–**d**) represent the dilution condition, while panels **e**–**h** correspond to the concentration condition. Predictions include the metagenome (**a**, **e**), both repressed and induced proteins (**b**, **f**), induced proteins (**c**, **g**), and repressed proteins (**d**, **h**). The color gradient represents data density, with yellow indicating higher densities and purple representing lower densities.

The enrichment of proteins with acidic residues is a well-documented adaptation to high salinity through the “Salt-in” strategy^6,8^. This phenomenon has been observed in metagenomes from saline and hypersaline environments^72–74^, leading to the hypothesis that similar patterns may emerge at the metatranscriptional level. To address this question, predicted proteins under salt dilution and concentration conditions were analyzed based on their molecular weight and isoelectric point (pI) (Fig. 8).

Overall, proteins with broad pI values dominated the predicted metagenomes (Fig. 8a, e), with most amino acids concentrated in the pI range of 4.0–6.0 and molecular weights below 50 kDa. However, a key difference was found in the repression of proteins with high pI values (> 9.0). Under salt concentration (Fig. 8h), these proteins exhibited stronger repression than under dilution (Fig. 8d), suggesting selective suppression of basic proteins in high-ionic environments. Moreover, the AB ratio (acidic amino acids Glu and Asp to basic amino acids Lys, His, and Arg)^74^ remained nearly identical between induced and repressed genes during dilution (1.391 vs. 1.389), indicating no strong selection for acidic proteins despite *Euryarchaeota* dominance. In contrast, the concentration experiment revealed an increase in induced genes encoding acidic proteins (AB ratio = 1.196), whereas repressed genes maintained a balanced ratio (1.0). This suggests that proteins favoring acidic residues are more likely to persist or be induced under salt stress, consistent with previous findings on halophilic proteome adaptations in *Salinibacter* and other “Salt-in” strategists^72–74^. Also, the observed increase in acidic amino acids further supports the notion that this adaptation occurs rapidly after a salinity shift.

**Fig. 9:**
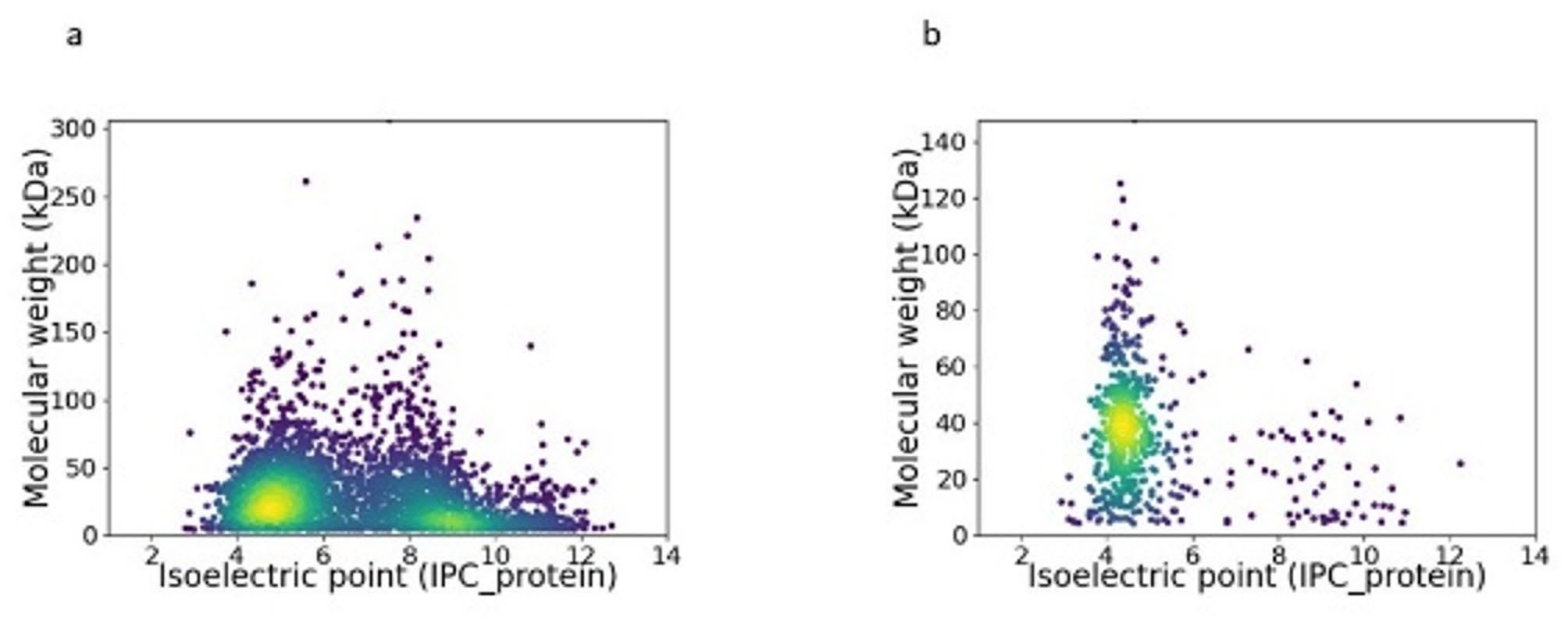
Distribution of repressed predicted proteins by taxonomic domain under high-salt concentration conditions in (**a**) Bacteria and (**b**) Archaea. The color gradient represents data density, with yellow indicating higher densities and purple representing lower densities.

A subsequent detailed analysis of the repressed proteins under high-salt concentration conditions further revealed domain-specific differences between Bacteria and Archaea (Fig. 9). Notably, Bacteria exhibited a higher overall number of repressed proteins compared to Archaea, and contributed significantly to the observed repression of basic proteins as a whole (Fig. 8). In bacterial predictions, the distribution spanned a broad range of isoelectric points—from highly acidic (~ 3) to strongly basic (~ 12)—with molecular weights extending up to approximately 250 kDa. In contrast, archaeal predictions were more narrowly distributed, with a predominant clustering between pI 4 and 6 and molecular weights generally below 140 kDa (Fig. 9).

These findings indicate that the overall repression of basic proteins in high-salt conditions appears to be largely driven by the bacterial fraction. This differential regulatory pattern underscores fundamental differences in the adaptive responses of Bacteria and Archaea, potentially reflecting evolutionary adaptations tailored to their distinct cellular architectures and ecological niches. The selective suppression of high pI proteins under increased salinity, reinforce the idea that salt stress drives adaptive changes in amino acid composition.

A potential limitation of our pI-based analysis is that posttranslational modifications (PTMs)—such as phosphorylation, acetylation, or methylation—can alter protein net charge in ways that in silico pI predictions do not capture. Indeed, our COG O data (Fig. 5a) show significant induction of genes involved in posttranslational modification in both bacteria and archaea under high salt stress, implying active PTM machinery that may shift actual protein pI away from predicted values. To address this, future studies will incorporate shotgun proteomics to directly identify PTMs and metabolomic profiling to identify modification substrates or byproducts (e.g., acetyl-CoA for acetylation), thereby enabling us to correlate transcript‐level PTM potential with observed changes in protein charge and function. This combined approach will refine our understanding of the “salt-in” strategy by revealing how PTMs modulate protein stability and activity in hypersaline habitats.

## Conclusion

This work provides a comprehensive metatranscriptomic evaluation of microbial adaptations to salt concentration and dilution, revealing that extreme osmotic stress triggers distinct transcriptional reprogramming in Archaea and Bacteria. Rather than simply reflecting shifts in community composition, our integrated analysis underscores that functional resilience—manifested through dynamic regulation of ion transport, stress responses, and metabolic pathways—predominates across divergent taxa. We propose a model in which halophilic Archaea, exemplified by dominant Euryarchaeota such as *Haloquadratum*, actively induce pathways related to osmoprotection, energy production, and ion homeostasis under high salinity and engage in broad transcriptional induction to sustain metabolic activity under stress. In contrast, bacterial populations predominantly conserve energy by downregulating growth-related functions while selectively activating stress-response genes, relying more on post-transcriptional and metabolic regulatory mechanisms. Additionally, the modulation of protein isoelectric points—with an enrichment of acidic residues and repression of basic proteins under high salt conditions, particularly in Bacteria—supports the “salt-in” strategy, ensuring protein stability and cellular functionality in extreme ionic conditions.

Our proposed model, which emphasizes differential adaptive strategies between Archaea and Bacteria may have significant implications for astrobiology. The observed functional resilience—despite shifts in taxonomic composition—suggests that extraterrestrial microbial life, if present in hypersaline environments such as those on Mars, Europa, or Enceladus, might be better detected through functional biosignatures rather than specific phylogenetic markers and provides a framework to predict how life could persist under extreme conditions.

Future research should aim to integrate metatranscriptomics with metaproteomics and metabolomics to establish direct links between gene expression and metabolic function. Also, investigating the temporal dynamics of these responses as well as the role of rare genes in osmoadaptation will offer deeper insights into our model. Ultimately, understanding these mechanisms will not only enhance our knowledge of microbial ecology in extreme environments but also further elucidate the molecular mechanisms underlying osmoadaptation and refine biosignature detection strategies in astrobiological exploration.

## Electronic supplementary material

Below is the link to the electronic supplementary material.

Supplementary Material 1

Supplementary Material 2

Supplementary Material 3

## Data Availability

Sequence reads generated in the present study were deposited in the NCBI SRA under the Bioproject accession number PRJNA595057.
