# Supplementary material for: Domain-specific osmoadaptation revealed by metatranscriptomic analysis in hypersaline environments
Source: Sci Rep. 2025 Jul 2;15:23320. doi: 10.1038/s41598-025-04148-4 (PMC12222773; doi:10.1038/s41598-025-04148-4)
Supplement: Supplementary file 3 — Supplementary Material 3 [file 41598_2025_4148_MOESM3_ESM.docx]

**Table S3**. Metagenomics assembly and metatranscriptomic sequences summary for samples C071 and CCAB.

|  | Concentration experiment (20% to 30%)  C071 | Dilution experiment (30% to 25%) CCAB |  |  |
| --- | --- | --- | --- | --- |
| METAGENOME  Total sequenced (Gpb) | 7,7 | 6,1 |  |  |
| Conting number | 34,964 | 38,699 |  |  |
| Mean sizes of the contigs | 2,592 | 2,449 |  |  |
| Contings range size | 1000-93,733 | 1000-191,930 |  |  |
| Gene number | 100,904 | 107,507 |  |  |
| METATRANSCRIPTOME |  |  |  |  |
| Total sequenced genes | 8,740 | 5,911 |  |  |
| Total differential expressed genes | 6,908 | 2,985 |  |  |
| Annotated Induced genes (>2) | 1,876 | 938 |  |  |
| Annotated Repressed genes (<2) | 1,736 | 774 |  |  |

**Table S4.** 2% of transcripts with significant highest induction after salt concentration experiment, sorted in order of expression level.

| COG group | Gene annotation | Taxonomic domain | Taxonomic genus | Fold Change |
| --- | --- | --- | --- | --- |
| O | *ATP-dependent Clp protease ATP-binding subunit ClpA* | Bacteria | *Paenibacillus* | 18,84826682 |
| O | *Heat shock protein* | Bacteria | *Flavobacterium* | 17,09274708 |
| P | *Sulfate permease or related transporter, MFS superfamily* | Bacteria | *Salinibacter* | 15,40605295 |
| C | *UPF0753 protein* | Archaea | *Salinarchaeum* | 15,30843247 |
| C | *NADH dehydrogenase subunit 5* | Archaea | *Halobacterium* | 15,27624482 |
| C | *Pyruvate:ferredoxin oxidoreductase or related 2-oxoacid:ferredoxin oxidoreductase, beta subunit* | Archaea | *Halorubrum* | 13,57177858 |
| O | *Molecular chaperone GrpE (heat shock protein)* | Bacteria | *Pelagibacteraceae (genus)* | 12,78896237 |
| P | *Sulfate permease or related transporter, MFS superfamily* | Bacteria | *Salinibacter* | 12,66546364 |
| P | *TonB-dependent Receptor Plug* | Bacteria | *Candidatus Methylomirabilis* | 12,62912567 |
| C | *Pyruvate:ferredoxin oxidoreductase or related 2-oxoacid:ferredoxin oxidoreductase, alpha subunit* | Archaea | *Halorubrum* | 12,26342863 |
| E | *Cysteine sulfinate desulfinase/cysteine desulfurase or related enzyme* | Bacteria | *Methylobacterium* | 11,79668815 |
| K | *DNA-binding transcriptional regulator, MarR family* | Bacteria | *Echinicola* | 11,48370423 |
| S | *Electron transport protein SCO1 SenC* | Bacteria | *Solitalea* | 11,19404642 |
| P | *Sulfate permease or related transporter, MFS superfamily* | Bacteria | *Salinibacter* | 11,17536684 |
| S | *integral membrane protein* | Archaea | *Haloquadratum* | 10,55870771 |
| C | *Ferredoxin* | Archaea | *Salinarchaeum* | 9,468027544 |
| S | *integral membrane protein* | Archaea | *Haloquadratum* | 9,234284296 |
| I | *Poly(3-hydroxyalkanoate) synthetase* | Bacteria | *Alkalilimnicola* | 9,204101501 |
| N | *Glycosyltransferase, catalytic subunit of cellulose synthase and poly-beta-1,6-N-acetylglucosamine synthase* | Bacteria | *Acetobacterium* | 8,800885979 |
| O | *Chaperonin GroEL (HSP60 family)* | Bacteria | *Psychroflexus* | 8,695082398 |
| IQR | *NAD(P)-dependent dehydrogenase, short-chain alcohol dehydrogenase family* | Archaea | *Haloquadratum* | 8,619188257 |
| S | Uncharacterized membrane protein YdjX, TVP38/TMEM64 family, SNARE-associated domain | Bacteria | *Rhodobacter* | 8,486191923 |
| S | prolyl-tRNA synthetase | Bacteria | *Nonlabens* | 8,455031014 |
| IQR | NAD(P)-dependent dehydrogenase, short-chain alcohol dehydrogenase family | Archaea | *Haloquadratum* | 8,358297088 |
| O | DnaJ-class molecular chaperone with C-terminal Zn finger domain | Bacteria | *Gramella* | 8,261609764 |
| E | Indole-3-glycerol phosphate synthase | Archaea | *Halopiger* | 8,197442834 |
| O | Chaperonin GroEL (HSP60 family) | Archaea | *Halovivax* | 7,83957854 |
| P | ABC-type nitrate sulfonate bicarbonate transport systems periplasmic components-like protein | Archaea | *Haloquadratum* | 7,775492489 |
| S | chad domain containing protein | Archaea | *Natrialba* | 7,58506376 |
| K | DNA-directed RNA polymerase, sigma subunit (sigma70/sigma32) | Bacteria | *Spiribacter* | 7,493707314 |
| J | Ribosomal protein S12 | Bacteria | *Mesotoga* | 7,355365151 |
| S | Protein of unknown function (DUF2795) | Bacteria | *Bdellovibrio* | 7,30455592 |
| T | Signal Transduction Histidine Kinase | Archaea | *Haloquadratum* | 7,186338232 |
| S | response regulator receiver protein | Bacteria | *Solitalea* | 7,156426224 |
| S | gas vesicle K | Archaea | *Natrialba* | 7,126003561 |
| P | ABC-type Mn2+/Zn2+ transport system, permease component | Bacteria | *Sorangium* | 7,083573204 |
| E | Diaminopimelate epimerase | Bacteria | *Sorangium* | 6,928160894 |

**Table S5**. 2% of transcripts with significant highest repression after salt concentration experiment, sorted in order of expression level.

| COG group | Gene | Taxonomic domain | Taxonomic genus | Fold Change |
| --- | --- | --- | --- | --- |
| G | *Citrate lyase beta subunit* | Bacteria | *Xanthobacter* | -24,27227088 |
| G | *extracellular solute-binding protein* | Bacteria | *Paracoccus* | -22,01167201 |
| E | *Alanine dehydrogenase* | Bacteria | *Xanthobacter* | -21,14434069 |
| G | *extracellular solute-binding protein* | Bacteria | *Dinoroseobacter* | -20,36855018 |
| H | *Cobalamin biosynthesis protein CobN, Mg-chelatase* | Bacteria | *Phenylobacterium* | -16,45740702 |
| J | *Ribosomal protein S4 or related protein* | Bacteria | *Candidatus Phytoplasma* | -16,32712449 |
| J | *Ribosomal protein L16/L10AE* | Bacteria | *Buchnera* | -15,92918332 |
| CP | *NADH:ubiquinone oxidoreductase subunit 5 (chain L)/Multisubunit Na+/H+ antiporter, MnhA subunit* | Bacteria | *Flavobacterium* | -15,84452947 |
| C | *NADH:ubiquinone oxidoreductase 27 kD subunit (chain C)* | Bacteria | *Buchnera* | -15,65966704 |
| J | *Ribosomal protein L6P/L9E* | Bacteria | *Candidatus Phytoplasma* | -15,06312262 |
| H | *Coproporphyrinogen III oxidase or related Fe-S oxidoreductase* | Bacteria | *Rhodobacter* | -15,05580016 |
| J | *Ribosomal protein S4 or related protein* | Bacteria | *Candidatus Phytoplasma* | -14,38333567 |
| G | *Abc transporter* | Bacteria | *Dinoroseobacter* | -14,24951882 |
| L | *Ribonuclease HI* | Bacteria | *Anaplasma* | -14,21731957 |
| E | *Indole-3-glycerol phosphate synthase* | Archaea | *Haloquadratum* | -14,01116904 |
| X | *Retron-type reverse transcriptase* | Bacteria | *Sphaerochaeta* | -13,22740664 |
| C | *NDH-1 shuttles electrons from NADH, via FMN and iron- sulfur (Fe-S) centers, to quinones in the respiratory chain* | Bacteria | *Buchnera* | -13,1222204 |
| M | *Protein of unknown function (DUF1611)* | Bacteria | *Rhodospirillum* | -12,80665805 |
| K | *DNA-directed RNA polymerase, beta' subunit/160 kD subunit* | Bacteria | *Bacillus* | -12,7027165 |
| K | *Leucine-rich repeat (LRR) protein* | Bacteria | *Enterococcus* | -12,59589988 |
| O | *ATP-dependent Clp protease ATP-binding subunit ClpA* | Bacteria | *Brevibacillus* | -11,80233556 |
| B | *Archaeal histone H3/H4* | Bacteria | *Anoxybacillus* | -11,7236393 |
| P | *Nitrogenase subunit NifH, an ATPase* | Bacteria | *Phenylobacterium* | -11,47912626 |
| J | *Ribosomal protein S8* | Bacteria | *Buchnera* | -11,47072867 |
| C | *subunit m* | Bacteria | *Buchnera* | -11,3983481 |
| R | *Tetratricopeptide (TPR) repeat* | Bacteria | *Bacillus* | -11,39430272 |
| C | *NDH-1 shuttles electrons from NADH* | Bacteria | *Staphylococcus* | -11,34762326 |
| C | *NADH:ubiquinone oxidoreductase 49 kD subunit (chain D)* | Bacteria | *Flavobacterium* | -11,34153455 |
| J | *Ribosomal protein L6P/L9E* | Bacteria | *Candidatus Phytoplasma* | -11,18915476 |
| C | *FoF1-type ATP synthase, alpha subunit* | Bacteria | *Flavobacterium* | -11,02696943 |
| P | *binding-protein-dependent transport systems inner membrane Component* | Bacteria | *Dinoroseobacter* | -10,59040624 |
| C | *NADH dehydrogenase/NADH:ubiquinone oxidoreductase 75 kD subunit (chain G)* | Bacteria | *Candidatus Profftella* | -10,56037697 |
| O | *Regulator of protease activity HflC, stomatin/prohibitin superfamily* | Bacteria | *Brevibacillus* | -10,5289297 |
| L | *helicase* | Bacteria | *Bacillus* | -10,41459811 |

**Table S6**. 2% of transcripts with significant highest induction after salt dilution experiment, sorted in order of expression level.

| COG group | Gene | Taxonomic domain | Taxonomic genus | Fold Change |
| --- | --- | --- | --- | --- |
| P | *Ammonia channel protein AmtB* | Archaea | *Halobacterium* | 34,90881456 |
| K | *Predicted transcriptional regulator, contains C-terminal CBS domains* | Archaea | *Natronomonas* | 22,77453621 |
| P | *Ammonia channel protein AmtB* | Archaea | *Halorubrum* | 22,69662187 |
| TE | *Nitrogen regulatory protein PII* | Archaea | *Halobacterium* | 20,91745939 |
| P | *Ammonia channel protein AmtB* | Archaea | *Halobacterium* | 17,47005243 |
| P | *Ammonia channel protein AmtB* | Bacteria | *Gordonia* | 16,33931002 |
| E | *Amino acid transporter* | Archaea | *Haloarcula* | 16,21968665 |
| J | *Ribonuclease G or E* | Archaea | *Natronomonas* | 15,88174111 |
| C | *Key enzyme in the regulation of glycerol uptake and metabolism (By similarity)* | Bacteria | *Marinobacter* | 15,51110894 |
| HR | *Flavin-dependent oxidoreductase, luciferase family (includes alkanesulfonate monooxygenase SsuD and methylene tetrahydromethanopterin reductase)* | Archaea | *Halalkalicoccus* | 13,83209637 |
| E | *Nitrogen regulatory protein pii* | Bacteria | *Gordonia* | 13,31381533 |
| V | *Enamine deaminase RidA, house cleaning of reactive enamine intermediates, YjgF/YER057c/UK114 family* | Archaea | *Haloquadratum* | 12,6719896 |
| E | *amidohydrolase* | Archaea | *Halalkalicoccus* | 12,13119122 |
| E | *Indole-3-glycerol phosphate synthase* | Archaea | *Haloquadratum* | 12,12950706 |
| E | *Extracellular solute-binding protein, family 5* | Archaea | *Haloquadratum* | 12,04364714 |
| P | *Trk K+ transport system, NAD-binding component* | Archaea | *Halobacterium* | 11,32890264 |
| P | *ABC-type phosphate transport system, periplasmic component* | Archaea | *Halorubrum* | 11,28792377 |
| Q | *alkaline phosphatase* | Archaea | *Halogeometricum* | 10,81267437 |

**Table S7**. 2% of transcripts with significant highest repression after salt dilution experiment, sorted in order of expression level.

| COG group | Gene | Taxonomic domain | Taxonomic genus | Fold Change |
| --- | --- | --- | --- | --- |
| C | *DNA-binding transcriptional regulator, Lrp family* | Archaea | *Natrinema* | -11,60502953 |
| D | *alkyl hydroperoxide reductase Thiol specific antioxidant Mal allergen* | Bacteria | *Salinibacter* | -10,97054367 |
| E | *peptidase s9 prolyl oligopeptidase active site domain protein* | Bacteria | *Salinibacter* | -8,663195692 |
| F | *Histidine kinase* | Archaea | *Natrinema* | -5,235034835 |
| J | *TonB-dependent Receptor Plug* | Bacteria | *Nitrospira* | -4,983023721 |
| K | *Alanyl-tRNA synthetase* | Archaea | *Haloquadratum* | -4,723554518 |
| O | *Uncharacterized membrane protein YadS* | Archaea | *Haloquadratum* | -4,717274745 |
| P | *Adenylate cyclase class IV, CYTH domain (includes archaeal enzymes of unknown function)* | Archaea | *Haloquadratum* | -4,609702162 |
| P | *Isocitrate dehydrogenase* | Archaea | *Haloquadratum* | -4,315282503 |
| S | *Essential cell division protein that forms a contractile ring structure (Z ring) at the future cell division site. The regulation of the ring assembly controls the timing and the location of cell division. One of the functions of the FtsZ ring is to recruit other cell division proteins to the septum to produce a new cell wall between the dividing cells. Binds GTP and shows GTPase activity (By similarity)* | Archaea | *Haloquadratum* | -4,267231309 |
| T | *Hypoxanthine-guanine phosphoribosyltransferase* | Archaea | *Haloquadratum* | -4,249074619 |
| T | *tonB-dependent Receptor* | Bacteria | *Salinibacter* | -4,156688414 |
| T | *CBS domain* | Bacteria | *Salinibacter* | -4,121030341 |
| TR | *GAF domain* | Archaea | *Natrinema* | -4,062538541 |
| V | *ABC-type multidrug transport system, ATPase and permease component* | Bacteria | *Salinibacter* | -4,04089957 |
